# Swimming Together Upstream: How to Align MLP Services with U.S. Healthcare Delivery

**DOI:** 10.1017/jme.2023.163

**Published:** 2023

**Authors:** William M. Sage, Keegan D. Warren

**Affiliations:** 1: TEXAS A&M UNIVERSITY SCHOOL OF LAW, FORT WORTH, TX, USA; 2: TEXAS A&M UNIVERSITY SCHOOL OF MEDICINE, FORT WORTH, TX, USA; 3: TEXAS A&M UNIVERSITY HEALTH SCIENCE CENTER, FORT WORTH, TX, USA.

**Keywords:** Medical-Legal Partnership, Value-Based Care, Social Determinants of Health, Social Needs Screening, Information-Sharing, Alternative Payment Methods, Z Codes, E/M Codes

## Abstract

Medical-legal partnership (MLP) embeds attorneys and paralegals into care delivery to help clinicians address root causes of health inequities. Notwithstanding decades of favorable outcomes, MLP is not as well-known as might be expected. In this essay, the authors explore ways in which strategic alignment of legal services with healthcare services in terms of professionalism, information collection and sharing, and financing might help the MLP movement become a more widespread, sustainable model for holistic care delivery.

Medical-legal partnership (MLP) is an established and successful model for delivery of healthcare in the U.S. Yet, it is not commonly found in the healthcare vernacular, even among medical professionals and policymakers dedicated to improving the health of vulnerable communities and populations. Consider, for example, that MLP is absent from most governmental healthcare frameworks. The Health Resources and Services Administration (HRSA) stands alone in clearly, expressly incorporating legal care into its conceptualization of health services — and it did so only in 2014.^1^ Even with the current proliferation of standards for entities to engage in remediating social determinants of health or improving health equity — goals that MLP demonstrably achieves^2^ — not one accrediting body has included integrated legal services as a mandate, or even a suggestion.^3^

The overtly legalistic phrase “health justice” — which both of us consider a more accurate description of the necessary restructuring and advocacy than “determinants,” “disparities,” or even “equity” — may eventually lead MLP to a more prominent position in health system improvement, but it has yet to do so. As we discuss below, there are both discordances and concordances between how MLP thinks of itself as a field and how the health care system operates.

In this article, we consider how attention to aligning the MLP model with traditional medical care financing and delivery might make more prominent the value of lawyers as onsite healthcare practitioners. Following a brief description of the professional resonance between MLP and the established medical sector, we organize the article around three themes: the way professionals move (clinical alignment); the way data move (informational alignment); and the way money moves (payment alignment).

In this article, we consider how attention to aligning the MLP model with traditional medical care financing and delivery might make more prominent the value of lawyers as onsite healthcare practitioners. Following a brief description of the professional resonance between MLP and the established medical sector, we organize the article around three themes: the way professionals move (clinical alignment); the way data move (informational alignment); and the way money moves (payment alignment).

Taken together, these forms of alignment represent levers to increase the sustainability and prominence of medical-legal partnership.

## MLP’s Health Equity Advantage: Professional Resonance in Fulfillment of Social Needs

It is challenging to integrate social care into the delivery of U.S. health care when, broadly construed, public support for the two sectors has been optimized through regulation for almost diametrically opposed values and approaches. American medicine is technological, individual, and entrusted to elite professionals through “entitlement,” while social services are largely anonymous, commoditized, and subject to the vagaries of tax bases and public budgeting procedures.^4^ Moreover, the U.S. divides its health-directed investments between the sectors inversely to the rest of the industrialized world, spending less than 60¢ on social services focused on wellness for every $1 spent on medical care in response to illness.^5^ Because 60-80% of health is driven by the latter, that inversion is likely a primary factor in our shorter, sicker lives.^6^

MLP is an exception to much of the rest of the social sector in that both its central components — law and medicine — are built around professional services. This approach to patients’ health-related social needs is potentially more intuitive to medical professionals than other models. Both the legal profession and the medical profession routinely identify problems for patients or clients and perform tasks, either personally or by collaboration and referral, intended to solve them. Both professions categorize and code their assessments (diagnoses) as well as their interventions, compile longitudinal records of services delivered, and receive compensation accordingly. And both law and medicine are largely self-regulating, with their value and values idealized in ethical schema, bounded by disciplinary codes, and evidenced by payment models.

MLP therefore may seem less “squishy” to physicians, other established health professionals, and healthcare organizations than many other health system forays into equity, particularly when services are delivered through multidisciplinary teams that collaborate to identify and address the social determinants of poor health.^7^ Through MLP, public interest lawyers can help operationalize the goals of physicians seeking to extend the measurable benefits of their expertise upstream from hospitals and clinics into community-based settings. Lawyers are not the only professionals working in those spaces, but there can be little doubt that law is a fundamental tool for addressing and organizing health-related social needs.^8^ There is, moreover, considerable commonality between the individuals and families served by (non-corporate) lawyers and those served by physicians, including common challenges of access to affordable services.

Similarly, health professionals concerned with health equity may find attractive the opportunities that MLP offers to change social and economic conditions at a macro level so as to benefit the health of many people at once.^9^ Moving “from patients to policy” aligns well with the public benefit aspects of legal ethics, which however committed to zealous advocacy on behalf of individuals also understands the importance of collectively challenging inequity caused by powerful governmental or corporate actors.^10^ Medical ethics, by contrast, has tended to see professional charity and advocacy mainly in terms of increasing direct access to individual clinical care or to insurance coverage for those services, although recent commentary seeks to broaden that perspective by focusing physicians on the moral determinants of health, including injustice.^11^

A misperceived clash of professional values presents a minor obstacle to the proliferation of MLP. Notwithstanding a lengthy history of collaboration between medical and legal professionals to address human and civil rights abuses, early MLP programs got underway in decades where antipathy between physicians and attorneys, generally around medical malpractice litigation, made the notion of partnership between the two professions newsworthy.^12^ Persistence of this “man bites dog” perception may be one factor limiting MLP’s prevalence.^13^

For their part, legal aid lawyers may also regard favorably the potential that partnering with physicians and hospitals in MLP offers for upstream impact. Although American medicine has long been criticized as overly specialized and reactive — a sick care system rather than a health care system^14^ — the situation is arguably worse for civil legal aid to the poor. Legal aid lawyers often find themselves understaffed, under-resourced, and trapped offering only stopgap, downstream assistance.^15^ As a result, access-to-justice studies routinely conclude that the legal system needs to be more effective in reaching vulnerable populations and using law preventively.^16^

Legal assistance that is fundamental to health has suffered from ideological targeting through the appropriations process; adjusted for inflation, the current budget for the Legal Services Corporation is less than one-third of what it was in 1980,^17^ and the Trump Administration routinely proposed zeroing out funding for legal services.^18^ It is in large part the recognition of public interest lawyering as countering majoritarian impulse, empowering those disadvantaged by the establishment, and potentially promoting redistribution of resources that exposes civil legal aid to constant fiscal-political constraint.^19^

MLP dangles the possibility of much more generous financial support, with fewer political constraints on the use of public funds^20^ and with greater deference by other parts of society to the reinforcing power of physicians’ opinions and determinations.^21^ In part for this reason, the American Academy of Arts and Sciences has identified MLP as “the most promising model” for collaborative redress of the justice gap, its metric for assessing the unmet need for civil legal services.^22^

Because MLP is able to leverage legal expertise to address health-related social needs at the individual, institutional, and systems levels, it is also a promising mechanism for orienting health professionals toward equity and justice. For example, interprofessional exchange of information and perspective has long been a core component of MLP, but — in a nod to lawyers’ superior structural expertise — it has evolved from clinicians training lawyers about medicine to lawyers training clinicians about the levers of social change. In its most effective iterations, the MLP model of healthcare delivery embeds lawyers in the clinic or hospital setting as specialized members of interdisciplinary care teams, going beyond referrals to collaboration and integrated service.^23^ Lawyers’ ability to interrogate social structures in pursuit of justice is the critical additional lens that MLP brings to health equity.

## The MLP “Valley of Death”: Clinical Compatibility, Information Exchange, and Sustainable Funding

Sustainability has been the principal challenge for health systems seeking to integrate MLPs, notwithstanding their relatively long history and growing professional resonance. Much as entrepreneurial ventures in industry often fail in a “valley of death” between start-up investment and market maturity, MLPs launched as exciting collaborative projects may not survive if either legal services organizations or clinical enterprises face unexpected financial difficulties or experiences changes of leadership and mission. These experiences in turn may make organizations reluctant to invest again in new programs.

This is not unique to MLP: many worthy projects in US health care die shortly after the special funding that initiated them runs dry. Although there is often a theoretical “business case” for sustainability based on projected clinical savings to hospitals or health insurers, in practice the business case frequently evaporates unless fee-for-service revenue becomes available. Even widespread enthusiasm for “hot-spotting” — intensive interventions to improve health for “super-utilizers” of emergency and inpatient services — was eventually tempered by statistical analysis challenging the viability of the business case.^24^

Health system revenue, and therefore workflow, is driven by payment for advanced clinical services to individual patients, which legal services often can supplement. Legal services, like medical services, generally follow a logical progression of professional diagnosis, treatment process, and measurable health outcome. It is uncommon, however, for the legal services and the medical services that can help establish the causes of ill health, address scientifically the health conditions identified, and prove effective for individual patient-clients in the real world to be catalogued and considered side-by-side.

This “operational crosswalk” between medical-clinical and legal-clinical interventions remains underspecified and underdeveloped, including how information is collected and transferred and what structural modifications are needed to accommodate legal professionals within the hospitals and clinics that employ or coordinate with physicians, nurses, and other health professionals. Even where a crosswalk may exist, such as an organ transplantation program or multidisciplinary clinic, it is typically not made explicit. As a result, MLP programs seem most compatible with novel re-conceptualizations of how the health care system should be organized and financed: universal coverage, full social service integration, and value-based payment. Few MLPs appear well adapted to the admittedly flawed ways in which trillions of medical dollars flow in the United States today.

If health policy advocates want to see MLPs thrive, so that the MLP model can help improve health equity and health justice in more sweeping ways over the longer term, those advocates must give priority to getting MLPs reasonably aligned and fairly paid today. Put differently, MLP in our view will bring about the most radical change in health care if it is radical within the system, not outside of it.

### Clinical Alignment and Basic Service Documentation

Because information exchange, payment, and structural accommodation derive from it, clinical alignment is the most important path to express recognition that the legal care provided by MLP *is* health care. In fact, the provision of professional medical and legal services is similar. It begins when a person arrives with a story, the elements of which become “symptoms” in medicine and “facts” in law. Next, using training, experience, and other tools of their respective trades, the medical or legal professional diagnoses a need and determines that a path exists for remediation or mitigation. When the professional establishes a fiduciary relationship with the person, memorializing the asymmetry resulting from the professional’s expertise and the person’s vulnerability, it subjects the services provided or omitted to review for adherence to professional standards. Whether the need evidenced by the presentation is resolved or not, the relationship typically concludes after provision of services. In the case of law, this is often formally acknowledged; regardless, however, it generally terminates the professional duty.

These similarities enable MLPs to crosswalk the medical services indicated for common clinical conditions (asthma, diabetes, sickle cell disease, cancer, etc.) with related legal services, noting the latter’s direct effect or reasonably anticipated incidental benefit. After all, research has shown that, throughout the lifespan from newborns to adults of advanced age, MLP has a positive health impact on a variety of clinical conditions affecting specific patient populations.^25^ The I-HELP mnemonic, which is used widely in MLP models to demonstrate to healthcare providers the range of potential health-harming legal needs,^26^ provides a framework for clinical cross-walking, as shown in Table 1.

**Table 1:** Clinical cross-walking through the I-HELP framework

Health-harming Legal Need	Sample Legal Intervention & Outcome	Sample Target Population
**Income &** **Insurance**	Representation at administrative hearing leads to recovery of denied SNAP benefit that increases access to nutritious food	Youth with diabetes^27^
**Housing**	Negotiation on behalf of tenant leads to remediation of mold and mildew	Children living in rental housing with asthma^28^
**Education &** **Employment**	Advocacy at Individualized Education Plan meeting leads to appropriate tools and goals in student’s plan	Youth with learning disabilities^29^
**Legal Status**	Petition to expunge or seal criminal history leads to employability	Peer support workers with substance use disorder^30^
**Personal & Familial Stability**	Counseling a family on alternatives to guardianship leads to retained legal personhood and healthy decision-making	Young adults who are neuroatypical^31^

Clinical pathways also exist beyond individual direct service. MLP lawyers contribute meaningfully to other clinical activities such as case conferences for complex patients,^32^ group medical visits for pregnancy or other routinized^33^ or non-routinized^34^ care, joint professional visits,^35^ and community health events.^36^ Institutional committees, workgroups, and task forces to develop or refine policies similarly may benefit from professionals with patient-centered legal skills; for instance, during a three-year period, one MLP catalyzed 19 community changes and 8 organization changes.^37^ Each of these collaborations increases professional interdependency and alignment in support of whole-person care.

Documenting legal services similarly to clinical services in a patient-client’s medical record is important, so that wherever an MLP is housed in the health care system, routine screening and treatment for associated health-harming legal needs become part of clinical protocols.^38^ As with medical interventions, legal interventions for a given condition can be targeted to those most at risk and prioritized for those most likely to benefit from them. Over time, moreover, documentation of legal interventions at the patient level can lead to improved processes at the population or institutional level if an issue is pervasive for a population.^39^

Consistent documentation can help identify new clinical crosswalks based on the health needs of the area served by the clinic or hospital, allowing the MLP to stratify the patient population for additional MLP evaluation and intervention. Segmentation strategies also enable MLPs to multiply the frontline capacity of the legal team by tailoring service delivery to each defined group.^40^ While legal services have always used conceptually similar approaches, often through financial, issue, or geographic eligibility requirements, clinical alignment requires being deliberate about language and expressly identifying legal care as an individual and population health strategy.^41^

The goal of clinical alignment suggests that the MLP legal team should be treated as any other consulting specialist and should have read/write access to the clinical host’s electronic health record (EHR). (We discuss the ethics and legality of bidirectional information-sharing below.) Informational alignment through the EHR is valuable to MLPs for several reasons. Bidirectional information flow between the legal and medical records helps the legal team, who frequently use medical evidence to further a patient’s legal claim.^42^ Collectively, linking the legal team’s data with the medical record also can reveal correlations between unmet individual needs and medical conditions, which can further population health management.^43^ More generally, the social service sector is chronically under-resourced in terms of technology, and can benefit from the extensive investments that the health care system has made in informatics.^44^

The easiest methods for accomplishing MLP-EHR linkage are often those that build on existing processes, and the host entity’s approach to integrated behavioral healthcare may offer a compatible model. Regardless, the MLP should map how referrals from clinicians to lawyers or other specialized professionals are made, how those appointments are tracked, and what constitutes closing the loop between specialized legal professionals and the referring physician after an initial consultation or following delivery of legal services. Loop-closure is important because clinical alignment requires clinician buy-in: What feedback will help clinicians regard the legal team as part of the health care team?

### Information-Sharing Between Legal and Medical Service Providers

Delivery of integrated care requires information-sharing at the patient/client level, and distinct professional values, technology, and privacy regulations complicate information-sharing. Identifying and managing the risks of informational alignment therefore imposes a significant compliance burden on both legal services organizations and their clinical hosts. Because different terms are used in health care (generally, “protected health information”) and in law (generally, “confidential (legal) information”), we use “personally identifiable information” (PII) as a collective term for both.

The medical partner is almost certainly a covered entity under the data privacy, security, and other aspects of federal Health Insurance Portability and Accountability Act (HIPAA) regulations as well as under state medical records privacy law.^45^ Additionally, special federal protections under 42 CFR Part 2 may be applicable because many healthcare entities are federally assisted and hold themselves out as providing treatment for substance use disorder (SUD), or are lawful holders of SUD treatment information.^46^

The legal partner will rarely be a HIPAA-covered entity on its own, but it will often have a business associate agreement that subjects it to HIPAA requirements.^47^ It also may be a covered entity under state medical records privacy law. Regardless, it is subject to state bar confidentiality rules.

Broadly speaking, PII within an MLP setting may be used or disclosed only with patient/client consent unless an exception applies.^49^ Because each partner is acting in furtherance of the patient-client’s goals and interests, and opportunities arise frequently to discuss contemplated uses and disclosures and, most critically, to obtain informed consent from the patient/client to whom the information belongs, bidirectional information-sharing is not inherently problematic. Notwithstanding subtle differences in conceptualizations of the patient-client between the two professions, moreover, treating everyone served with respect and dignity is a common ethical priority. Whether or not formal written consent to use or disclosure of PII is required by the partnering entities, patient-client control over their information is part of centering that individual in the professional relationship.

By mapping incidents and patterns of PII use and disclosure, inter-professional MLP teams can incorporate technological constraints and applicable legal-regulatory requirements into their designed workflow, and can identify informational decision points for each partnering entity.^50^ Table 2 shows representative decision points derived from the National Center for Medical-Legal Partnership’s workflow diagram for referrals between partnering entities; fully integrated MLPs may have different or additional decision points.^51^

**Table 2:**
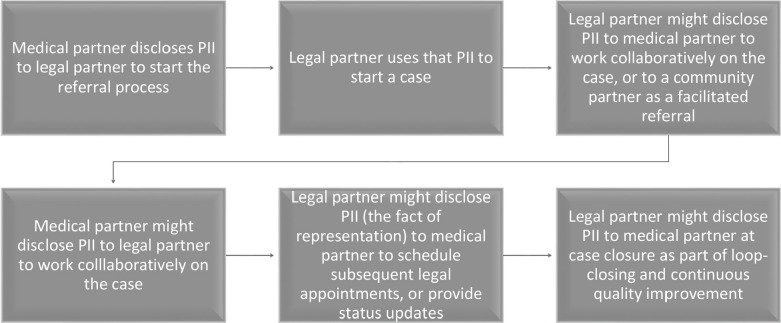
Decision points for information-sharing in MLP

### Tasking and Coding MLP Services in Clinical-Administrative Data Systems

Clinical documentation and ethical, legally permissible information-sharing within the patient-client record is necessary for informational alignment in MLP, but it is not sufficient. For better or worse, the U.S. health system collects, categorizes, and transfers information primarily in order to be paid for services delivered, generally through claims filed with private and governmental health insurance. Currently, even MLPs that utilize the health partner’s EHR to manage referral work may stop there, having successfully used newly available, well-funded technology to connect data. But technology is only a tool, and a referral platform is not a solution to MLP sustainability. An informational pathway that ends with the patient-client record may not generate sufficiently robust medical funding to address health-harming legal needs.

For MLP, fundability also requires translating discrete clinical assessments and subsequent interventions into tasks (whether screening, “evaluation and management,” or specialized service delivery) with codes appropriate for generating payment, consistent with the clinical approach to documentation. This structured clinical-administrative data may include modifiers to codes for medical interventions that improve access or enhance payment by virtue of legal evaluation. Structured data is also essential for research use, including by academic investigators who may be part of the MLP’s clinical host entity.

Fortunately, legal aid organizations already generate and use structured data, documenting legal interventions using an electronic case management system. LegalServer, the most popular such system, includes fields targeted to several key functions of MLP legal teams:^52^

- Recording referrals from healthcare partners, using Prescreens and Callbacks
- Using branch logic to ask MLP questions during intake
- Adding and updating MLP information during a case
- Viewing MLP info on special MLP tabs in a Case Profile View
- Collecting MLP information on case closure
- Tracking MLP Consults & Referrals
- Running reports with MLP-specific information

As noted, legal services are similar to medical services in that they include professional diagnosis, intervention, and outcome. Through relatively simple modification of backend tables in the EHR, these information elements of MLP legal services can be recorded in appropriately structured data fields to gain financial support that other social needs care may not. At least one MLP has succeeded in automating bidirectional data-sharing between the EHR and LegalServer using application programming interfaces (API), although another effort was unsuccessful because of EHR interoperability difficulties within the clinical network and limited clinic-side human resources.^53^ If automated processes are too challenging technically, manual entry remains possible. Regardless, MLPs must make an effort to place legal services data on an equivalent footing with healthcare services data. As discussed in greater detail below, recognizing operationally that a primary purpose of healthcare data is to bill for the services provided opens significant new pathways to financial sustainability for MLPs.

### Screening for Social Needs

Screening for relevant conditions is a common, intuitive practice in both medical care and public health that readily translates to other social circumstances, whether to quantify aggregate burden, identify individual or community need, or track progress. Screening has been an essential aspect of alerting the health care system to racial and ethnic disparities in illness, access to treatment, and outcomes, and in reorienting the health care to addressing social determinants of health and achieving health equity.

Some screening tools, such as PRAPARE, have been designed to determine the existence of a health-related social need.^54^ As with other forms of medical screening, a patient can screen positive and, in theory, receive an appropriate intervention. Unfortunately, however, evidence connecting commonly used social screening methods to interventions producing measurable health improvement is, at best, nascent.^55^ Moreover, these tools tend to avoid the most compelling aspects of the medical model: clear diagnostic criteria for the need, appropriate triage for addressing one person’s need versus another’s, tiered coding reflective of the complexity of the need, individualized intervention according to professional standards, and horizontal integration of the process throughout the delivery system.

In recent years, governmental entities including CMS, accrediting organizations such as the Joint Commission, private payers, and others have built health-related social screening requirements and associated data-collection obligations into their standards, conditions of participation, and supplemental payment policies (including risk adjustment) for health care providers. MLPs can enhance the utility of these requirements by leveraging their measurable, evidence-based screening practices that clearly distinguish longstanding, typically aggregate “determinants” from individually addressable “needs” that can be timely met. MLPs also can be deliberate about aligning the provision of legal services with the provision of medical services, building relationships beyond simple referral, and fostering mutual understanding and teamwork.

Social needs screening tools are seldom written with the problem-solving mindset that an experienced MLP legal team employs.^56^ Using skills similar to those needed to conduct intake for a new client, the MLP legal team can help generate action-oriented screening questions that enable meaningful tracking of service effectiveness rather than merely data mining demographic characteristics or risk factors. Such activity is consistent with MLP becoming a catalyst for institutional change, as discussed above. Moreover, a legal intake is based on fact-gathering that matches legal standards in much the same way that a medical intake is based on symptom assessment that matches diagnostic criteria. Consequently, MLP legal teams can help align a clinic or hospital’s social needs screening with its clinical screening.

Social needs screening tools do not necessarily prioritize limited resources. A triage process is as appropriate for social needs as for medical needs, and social needs are seldom emergent. For those whose income and resources are modest, a diet of processed foods or a lack of assured housing beyond the next paycheck may be normal, albeit undesirable. The MLP legal team is adept at prioritizing health-related social needs and determining the timeline on which a given need can be remediated.

Additionally, social needs screening tools typically treat need as one-size-fits-all within broad domains of social circumstances, either because the range of interventions may not be known to the designers of the questionnaire, or because the healthcare entity has limited relationships with the social services sector and thus can provide only limited responses. Prevalent screening methods may therefore overlook the fact that social needs (and social assets) manifest differently for each individual or family, which is a significant departure from the customization of need and response that characterizes medical practice.

By contrast, an effective MLP screening system incorporates the professionally appropriate, tiered scope of service for each identified legal need,^57^ which also can facilitate population health management and continuous quality improvement activities. As physicians anticipate potential medical interventions, MLP screening for potential legal interventions can distinguish between services that are quickly provided to resolve a minor acute need, such as offering basic advice, and ongoing assistance with a chronic need that requires sustained investment of resources, such as litigation. MLP legal interventions with greater intensity and/or duration can be consistently recorded, appropriately coded, and tracked in the case management system, whether or not they are presently eligible to be submitted for payment.

### Filling in the “Last Mile” of MLP Operations so That Money Flows

Absent a consensus, backed by workflow equivalence, that legal care for health-harming legal needs *is* health care, it should come as no surprise that adequate, reliable funding remains the biggest barrier to sustainability, and hence proliferation, of the MLP model. As explained below, there seem to be two pathways by which MLP can generate billable outcomes: charging for clinically beneficial services provided to individuals, and helping healthcare organizations meet institutional goals or requirements involving health equity.

Sources for MLP financing typically include legal fellowships, federal, and state legal services dollars, project funding through Medicaid waivers, HRSA funding of Federally Qualified Health Centers (FQHCs), hospital community benefit dollars, administrative line items, and, in the academic space, universities.^58^ A 2019 national environmental scan found that the median MLP budget is a paltry $100,000 annually, typically cobbled together from multiple sources, which supports a median of 1.0 FTE of attorney time and 0.2 FTE of support staff.^59^ Because of unreliable funding, MLPs may operate with a skeleton legal staff, which disrupts programmatic integrity in the same way that other aspects of philanthropy-driven social services frequently are forced to survive on a grant-to-grant, competitive basis.

As policymakers’ attention focuses on health equity, MLPs face an important strategic choice. One option is to pursue payment for legal services in conceptual parity with payment for medical services. As a practical matter, the best assured source of sustainable funding would be MLP payment on a fee-for-service basis in most settings and for most services, which is still an uphill climb in terms of political advocacy and payer acceptance. As a policy matter, MLP functions eventually should be expressly brought within an integrated, value-based financial model that would also apply to clinical care. Some MLPs have already piloted so-called Alternative Payment Methods (APMs), such as participation in regional accountable care organizations serving public and/or private health insurers.^60^

The second option is to seek sustainability mainly through supplemental payment (either directly or as a clinical enhancement) based on MLP facilitation of compliance by a hospital or clinic with a health equity mandate or an equivalent demonstration of value to low-income or minoritized communities. One vehicle used by MLPs partnering or integrated with tax-exempt hospitals — still the most common model — is measurable compliance with legal expectations regarding community benefit, which have been made more visible and concrete through required Community Health Needs Assessments (CHNAs) under the Patient Protection and Affordable Care Act.^61^ For MLPs associated with Federally Qualified Health Centers, HRSA funding for case management and enabling services, both of which expressly include legal services, may bring sustainability within reach, as health centers with MLPs typically have larger budgets than health centers without MLPs.^62^

Similar eligibility for public funding of health-improving legal (and other social) services has been granted under federally negotiated Section 1115 demonstration project waivers at the state level, payable through both Medicaid managed care plans and supplemental DSRIP allocations.^63^ In January 2023, California became the first state with an approved waiver to provide re-entry services prior to a person’s release from prison,^64^ which may be a funding opportunity that spurs growth of carceral MLPs, as may the April 2023 decision to pilot the provision of civil legal services within select federal prisons.^65^ Expanded funding for behavioral health in many states is also a natural fit for MLP because of the close alignment between lawyers and social workers in serving persons with mental illness or substance use disorder.^66^

These waivers and initiatives have shown preliminary success and should be replicated. For example, evaluation of a per-member, per-month add-on for Medicaid managed care plans to provide enhanced care management in Colorado showed that patient-clients who received legal services reported improved physical and emotional health and had fewer ED and hospital admissions, fewer missed medical appointments, and fewer missed days of work.^67^

No matter which approaches are pursued in a given MLP setting, evidence-based coding and outcomes measurement will be necessary to make the case for sustained payment. As mentioned above, equity-focused accreditation standards increasingly require data collection, whether directed at health plans (e.g., NCQA, Leapfrog Group) or at hospitals (e.g., Joint Commission). MLP legal teams routinely gather much or all of this data from patients. Data that may be hard for a clinician to obtain, such as granular data about significant cultural or geographic differences among populations and individual, may be routine for a legal team working on health-harming legal needs. For example, Terra Firma, an MLP addressing asylum claims for unaccompanied minors, collects detailed race, ethnicity, intergenerational nationality, and household data because the applicable laws demand specificity. Those laws also require sophisticated evaluation of mental health needs with referrals from the legal team to the medical team.^68^

Emerging generations of physicians, nurses, and other health professionals appear receptive to making the health care system operate more ethically, and promoting change from within AHCs using the MLP model places health justice prominently in health professions training.

The benefits to patient-clients of these legal skills, and the quality of the data that result, should improve with integrated financing models. As MLP information practices mature, legal care data can help to guide appropriate CPT codes for reimbursing office visits and ED visits. For example, E/M codes 99204 and 99205 are currently used by clinicians to reflect moderate levels of medical engagement for a new patient, as are 99214 and 99215 for established patients. The American Medical Association (AMA), which produces the codes, has observed that unmet social needs may raise the risk of morbidity by significantly limiting diagnostic capacity and treatment options.^69^

An article on the AMA website is instructive: During the most recent CPT annual symposium, Margie Andreae, M.D., a member of the AMA/Specialty Society RVS Update Committee, gave the example of a young man whose low-paying job does not provide health insurance, causing him to decline an MRI and referral for knee injury.^70^ Because of that social determinant of health, she explains, the physician cannot obtain information necessary to managing the patient’s condition, raising the level of complexity in medical decision-making and thus the level of the E/M service. The same article quotes Nelly Leon-Chisen, the American Hospital Association’s Executive Director of Coding and Classification, suggesting that “with enough data on specific diagnosis codes, SDOH can eventually be considered to reflect higher severity and intensity of services that will result in additional coverage and reimbursement.” Information-gathering by MLP legal teams should help integrated providers both demonstrate the need for complex care and document the provision of appropriate, effective services.

In anticipation of future broadening of fee-for-service payment (or APM) eligibility, MLP teams also should engage in detail with the “Z codes” that already exist in the ICD-10 framework. Codes Z00-Z99 are provided for occasions when circumstances other than a disease, injury, or external cause classifiable to categories A00-Y89 are recorded as “diagnoses” or “problems.” A subset, Z55-Z65, are important for social care because they can be recorded when some circumstance or problem is present that influences a person’s health status but is not in itself a current illness or injury. Anyone can diagnose using those codes, and the MLP legal team will generally find them easy to apply, which should increase uptake by the medical care team as well. Moreover, a legal referral can be made in reliance on a Z code diagnosis, and legal intervention(s) and outcome(s) can be coded subsequently to close the care loop in consultation with the medical team.

Other billing codes may offer similar opportunities. One study has shown that advance care planning happens more competently and more frequently where there is an MLP,^71^ opening the possibility of reimbursable joint medical-legal visits under existing Medicare codes 99497 and 99498. These codes are expressly permitted to be billed when performed as team-based services that are ordered and managed by a participating clinician.^72^

All in all, use and additional development of these billing codes is an important step in furtherance of sustainable payment for MLPs. Over time, integrated MLP entities will be able to code in a way that includes a professional diagnosis of social circumstances, that describes the additional MLP care that the patient receives, and that ensures reimbursement commensurate with need and service.

## Conclusion: Advancing the Broader Vision Through Education and Generational Change

For MLP advocates, as for others pursuing fundamental aspects of health system improvement, success will not come without “constancy of purpose.”^73^ Greater clinical, informational, and financial alignment between legal and medical services can help MLP become a widespread, recognized, and sustainable aspect of healthcare delivery, and can reduce the risk that the larger movement in support of health justice and health equity turns out to be transitory or ineffective. But long-term incorporation of MLP into professional norms and patient expectations is essential as well.

One of the most significant recent developments in the MLP service model has been the greater involvement of academic health centers (AHCs), health professional training programs, universities, and graduate schools of law, social work, and other fields.^74^ Without idealizing academic medicine’s historical role in defining U.S. health care, this trend seems promising not only for MLP, but also for health justice, health equity, and the broader public interest.

Emerging generations of physicians, nurses, and other health professionals appear receptive to making the health care system operate more ethically, and promoting change from within AHCs using the MLP model places health justice prominently in health professions training.^75^ In addition, clinical care (with its associated revenue) is the life-blood of AHCs as well as non-academic health systems, so teaching the MLP model helps build interprofessional collaboration and change workflow within established institutions, which in turn reduces the harms caused by prior care practices and enhances the measurable benefits of attention to health equity. Finally, interdisciplinary research in AHCs can be expected to expand and improve the evidence base not only for MLP, but also for other methods of engaging communities and addressing health-harming needs without unduly medicalizing social problems. The strong commitment to justice as well as science among students and trainees today is an encouraging sign.

## Note

The authors have no conflicts to disclose.
